# Evaluation of the Efficacy of an S-INDEL PEDV Strain Administered to Pregnant Gilts against a Virulent Non-S-INDEL PEDV Challenge in Newborn Piglets

**DOI:** 10.3390/v14081801

**Published:** 2022-08-17

**Authors:** Loni Schumacher, Qi Chen, Lindsay Fredericks, Phillip Gauger, Meggan Bandrick, Marcia Keith, Luis Giménez-Lirola, Drew Magstadt, Wannarat Yim-im, Michael Welch, Jianqiang Zhang

**Affiliations:** 1 Department of Veterinary Diagnostic and Production Animal Medicine, Iowa State University, Ames, IA 50011, USA; 2 Zoetis, Kalamazoo, MI 49007, USA

**Keywords:** porcine epidemic diarrhea virus, PEDV, S-INDEL, non-S-INDEL, pregnant gilts, protection

## Abstract

A safe and efficacious live-attenuated vaccine for porcine epidemic diarrhea virus (PEDV) is not commercially available in the United States yet. Two major PEDV strains are currently circulating in US swine: highly virulent non-S-INDEL strain and milder virulent S-INDEL strain. In this study, the safety and protective efficacy of a plaque-purified S-INDEL PEDV isolate formulated as a vaccine candidate was evaluated. Ten pregnant gilts were divided into three groups and orally inoculated at 79 days of gestation and then boosted at 100 days gestation (T01: n = 4, vaccination/challenge; T02: n = 4, non-vaccination/challenge; T03: n = 2, non-vaccination/non-challenge). None of the gilts had adverse clinical signs after vaccination. Only one T01 gilt (#5026) had viral replication and detectible viral RNA in feces. The same gilt had consistent levels of PEDV-specific IgG and IgA antibodies in serum and colostrum/milk. Farrowed piglets at 3 to 5 days of age from T01 and T02 gilts were orally challenged with 10^3^ TCID50/pig of the virulent non-S-INDEL PEDV while T03 piglets were orally inoculated with virus-negative medium. T01 litters had overall lower mortality than T02 (T01 36.4% vs. T02 74.4%). Specifically, there was 0% litter mortality from T01 gilt 5026. Overall, it appears that vaccination of pregnant gilts with S-INDEL PEDV can passively protect piglets if there is virus replication and immune response induction in the pregnant gilts.

## 1. Introduction

Porcine epidemic diarrhea virus (PEDV), the causative agent of porcine epidemic diarrhea (PED), was first detected in Europe in the 1970s followed by detection in some Asian countries in the 1980s and thereafter [1,2]. The sporadic and/or endemic PEDV infections in these countries did not attract significant global attention until the emergence of a highly virulent PEDV strain in China in late 2010 [3]. In North America, PEDV was detected for the first time in United States swine in April 2013 [4] and was subsequently reported in Canada [5] and Mexico [6]. Since then, emergence or re-emergence of PEDV has been reported in Southeast Asia, Europe, and South America [1,7,8]. PEDV remains a significant challenge to global swine industries.

At least two genogroups of PEDV are widespread in the US as determined by spike (S) gene sequences: (1) G1b (S-INDEL) and (2) G2b (US prototype or non-S-INDEL). The US non-S-INDEL PEDV genetically resembles pathogenic strains of PEDV that emerged in China in 2010 [6,9]. The S-INDEL PEDV is a variant strain that appeared in 2014 in the US containing insertions and deletions (INDELs) in the S protein [10]. Pathogenicity studies have demonstrated that S-INDEL PEDV is less pathogenic than non-S-INDEL PEDV in suckling piglets [11,12,13] and in weaned (28 days old) pigs [14], yet these two virus strains have serological cross-reactivity and cross-neutralization in vitro [15,16]. In addition, an in vivo study by Goede et al. showed that sows exposed to an S-INDEL PEDV could provide partial protection to newborn piglets challenged with a US non-S-INDEL strain seven months later [17]. Similarly, Lin et al. demonstrated that neonatal piglets experimentally inoculated with an S-INDEL PEDV were partially protected against challenge with non-S-INDEL PEDV at 21–29 days post the initial exposure [13]. In addition, inoculation of two sows with a German S-INDEL PEDV isolate four weeks before farrowing led to protection of their offspring against challenge with the homologous S-INDEL PEDV [18]. These studies suggest that the less virulent US S-INDEL strain could be a good candidate for modified lived virus (MLV) PEDV vaccine development.

Neonatal piglets are most susceptible to PEDV infection and disease. Protection of piglets relies on acquiring PEDV-specific antibodies from colostrum/milk uptake during lactation. Therefore, it is critical to activate the sow’s gut-mammary gland-secretory IgA axis to provide lactogenic immunity to the piglet [19]. However, vaccination route is key to stimulate mucosal immunity. Currently in the US, there are two commercial PEDV vaccines for intramuscular administration into pigs: an inactivated whole virus vaccine from Zoetis and an RNA particle vaccine containing the PEDV spike protein from Merck (previously acquired from Harrisvaccines^TM^) [20,21]. Some studies [22,23] showed that these PEDV vaccines boost the immune responses in herds previously exposed to PEDV (live virus) but did not induce good IgA response in naïve pigs after vaccination, a drawback of the limited induction of mucosal immunity. Experiences with transmissible gastroenteritis virus (TGEV) suggest that oral administration of live-attenuated vaccine induced better lactogenic immune responses than intramuscular inoculations of the same virus in naïve sows [24,25]. This supports the thought that oral administration of the MLV PEDV vaccine is more efficacious than the killed or subunit vaccines for inducing mucosal immunity. However, such a safe and efficacious PEDV MLV vaccine against the emerging US strains does not currently exist in the United States. The objective of this study was to evaluate the safety of a plaque-purified S-INDEL PEDV cell culture isolate (USA/IL20697/2014) orally administered to gilts pre-farrowing and to further evaluate its passive protective efficacy to piglets against challenge with a virulent non-S-INDEL PEDV as determined by the parameters such as mortality, virus shedding, clinical signs, and specific production of PEDV antibodies.

## 2. Materials and Methods

### 2.1. Virus Isolates and Cells

The US S-INDEL PEDV isolate USA/IL20697/2014 was isolated in our laboratory previously [15]. The isolate was passaged in Vero cells (ATCC CCL-81) and then plaque-purified to generate the attenuated PEDV MLV product IVP (Zoetis serial # K1115LS22) that was used to inoculate pregnant gilts in this study. The US non-S-INDEL PEDV isolate USA/IN19338/2013, previously isolated in our laboratory [26] and demonstrated highly virulent in multiple pig studies [11,27,28], was used at the passage 7 to challenge piglets in this study. Infectious titers of PEDV inocula were determined in Vero cells (ATCC CCL-81) as previously described [26]. All isolates were confirmed negative for porcine deltacoronavirus (PDCoV), TGEV, porcine rotaviruses (A, B, C), porcine reproductive and respiratory syndrome virus (PRRSV), and porcine circovirus (PCV) 2 and 3 by respective PCRs.

### 2.2. Animal Experimental Design

The experimental protocol was approved by the Iowa State University Institutional Animal Care and Use Committee (IACUC 2-16-8193-S). Ten pregnant gilts at 74 days of gestation were purchased from a conventional breeding farm and housed at Iowa State University Livestock Infectious Disease Isolation Facility (LIDIF). Sows were fecal swab negative for PEDV, TGEV, PDCoV, and porcine rotaviruses (A, B, C) by virus-specific PCR and serologically negative for PEDV by indirect fluorescent antibody (IFA) assay and PEDV whole-virus ELISA antibody assay upon arrival.

**Figure 1:**
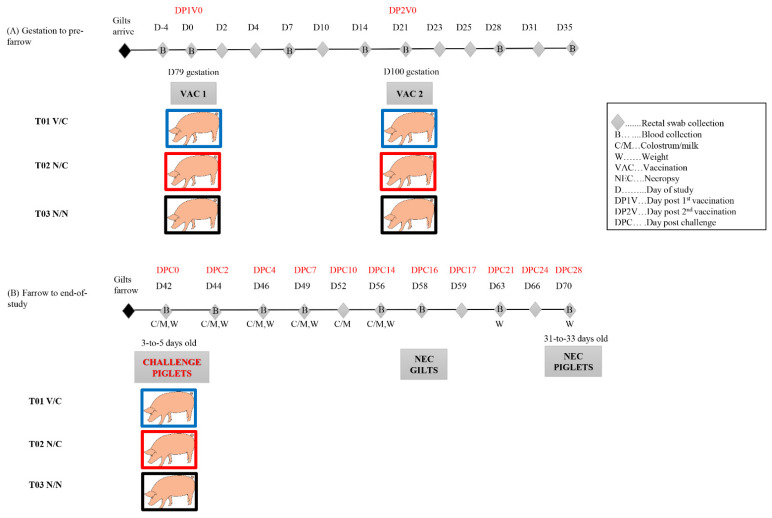
Timeline of sample collection during the major study events. The blue, red and black squares are used to distinguish the gilts in T01 V/C, T02 N/C, and T03 N/N groups.

As shown in Table 1 and Figure 1, gilts were randomly assigned to three treatment groups: T01 vaccination/challenge (V/C), n= 4; T02 non-vaccination/challenge (N/C), n = 4; and T03 non-vaccination/non-challenge (N/N), n = 2. Gilts were orally inoculated at 79 days of gestation (DP1V 0 corresponding to D0). T01 (V/C) was orally inoculated with the formulated US S-INDEL PEDV isolate USA/IL20697/2014 (target titer 10^4^ TCID50/mL, 10 mL/pig). T02 (N/C) and T03 (N/N) groups were orally inoculated with virus-negative culture media (10 mL/pig). Vaccination was repeated (boosted) three weeks later at 100 days of gestation (DP2V 0 corresponding to D21) with the same amount of the same inocula. Gilts were monitored for adverse clinical signs immediately after each oral vaccination and then daily to the end of the study. Gilts farrowed within three days of each other. Piglets were allowed to suckle colostrum and nurse from their dam where they were kept in individual farrowing crates with no cross fostering. At 3–5 days of age (DPC 0, corresponding to D42), piglets from T01 and T02 were orally challenged with 10^3^ TCID50/pig (5 × 10^2^ TCID50/mL, 2 mL per pig) of a highly virulent US non-S-INDEL PEDV isolate USA/IN19338/2013 at the passage 7. Piglets from T03 were orally inoculated with 2 mL of virus-negative culture medium. Piglets were monitored daily for adverse clinical signs throughout the study and weighed weekly starting from DPC 0. Diarrhea severity (fecal score) was determined with the following criteria: 0 = normal, 1 = soft (cowpie consistency), 2 = liquid with some solid content, 3 = watery with no solid content. Depression was scored with the criteria: 0 = normal (pig is up and active when entering the room or when approaching), 1 = mild depression (pig is slightly inactive, but gets up after being approached, pig moves slowly; pig shows some interest in food and social interaction and only stays active for a few minutes; animal may have rough coat), 2 = moderate depression (pig shows moderate inactivity and only gets up after moderate back pressure or stimulation; pig appears lethargic; pig displays little interest in surroundings food and/or social interaction; pig does not stay active long and may lie back down almost immediately; pig tends to stand with head up; pig has a rough coat; pig may stagger and appear slightly uncoordinated when walking), 3 = severe depression (pig shows pronounced inactivity and only gets up after pronounced back pressure or stimulation; pig shows pronounced lethargy; pig displays no interest in surroundings, food and/or social interaction; pig lies back down almost immediately and/or stands with head down; pig has a noticeably rough coat; pig staggers or has an uncoordinated gait; pig may appear anxious). For those piglets that died or met criteria for euthanasia (e.g., severe depression), a necropsy was immediately conducted. The remaining piglets were weaned at approximately 21 days of age (DPC 16; D58) and subsequently fed a pelleted starter diet ad libitum. All gilts were euthanized and necropsied when piglets were weaned (D58). All remaining piglets were euthanized and necropsied when they were at 28 days post challenge (DPC 28; D70).

Rectal swabs were collected from gilts at D(−4), D0 (first vaccination), D2, D4, D7, D10, D14, D21 (second vaccination), D23, D25, D28, D31, D35, D42 (piglet challenge day), D44, D49, D52, D56 and D58 (piglet weaning and gilt necropsy). Serum samples were collected from the gilts at D(−4), D0, D7, D14, D21, D28, D35, D42, D49 and D58. Colostrum/milk was collected from gilts at D36−D39 (farrowing days), D42, D44, D46, D49, D52, and D56. For piglets, rectal swabs were collected daily for the first week post challenge, then at DPC 10, 14, 17, 21, 24, and 28; serum was collected at DPC 0, 4, 7, 14, 21, and 28 (D42, D46, D49, D56, D63, and D70).

At necropsy, gilt and piglet enteric tissues were grossly examined by a pathologist blind to the study. Both fresh and 10% formalin-fixed small intestine, cecum, and colon tissues were collected for further PCR testing and histopathological examination, respectively.

### 2.3. Collection and Processing of Serum Samples and Colostrum/Milk Samples

Blood samples were drawn from the jugular vein of gilts and piglets at the indicated time points and processed by centrifugation at 1500× *g* for 15 min, aliquoted into 2 mL cryogenic tubes and stored at −20 °C until used. Colostrum and milk samples were collected from gilts at the indicated time points and processed by centrifugation at 13,000× *g* for 15 min at 4 °C to remove fat and debris. Then, the defatted samples were aliquoted and stored at −20 °C until tested.

### 2.4. Virus Shedding as Examined by a Quantitative PEDV Real-Time RT-PCR

Rectal swabs were collected from gilts and piglets at previously indicated time points and submerged into 1 mL phosphate buffered saline (PBS, 1X pH 7.4) immediately after collection, and stored at −80 °C until tested. Nucleic acids were extracted from rectal swabs (100 µL) using a MagMAX^TM^ Pathogen RNA/DNA Kit (Thermo Fisher Scientific, Waltham, MA, USA) and a Kingfisher-96 instrument (Thermo Fisher Scientific) following the instructions of the manufacturer. Nucleic acids were eluted into 90 µL of elution buffer. Primers, probe and in vitro-transcribed RNA used to generate standard curves of a quantitative PEDV nucleocapsid (N) gene-based real-time RT-PCR have been previously described [28,29,30]. Five microliters of each RNA template was used in a PCR setup in a 25 µL total reaction using the Path-ID Multiplex One-Step RT-PCR Kit (Thermo Fisher Scientific). Amplification reactions were performed on an ABI 7500 Fast instrument (Thermo Fisher Scientific) with the following conditions: 1 cycle of 48 °C for 10 min, 1 cycle of 95 °C for 10 min, and 40 cycles of 95 °C for 15 s and 60 °C for 45 s. The results were analyzed using an automatic baseline setting with a threshold at 0.1. Threshold cycle (C_T_) values < 40 were considered positive. Based on standard curves, the virus concentration (expressed as genomic copies per mL) in test samples was calculated. The mean virus concentrations were calculated based on all piglets within the group at each indicated time point (both PCR-positive and PCR-negative pigs).

Selected samples were also tested by a spike gene-based differential real-time RT-PCR, which can distinguish the US non-S-INDEL and S-INDEL PEDV strains. The primers and probes include: non-S-INDEL forward primer 5′-CAGTYGTTGYACTGGGCGGTTAT-3′, non-S-INDEL reverse primer 5′-CATGAACGCCACTAGCAGTTG-3′, non-S-INDEL probe 5′-FAM/CAATTCAACTTGGTACTGTGC/MGB-3′, S-INDEL forward primer 5′-TGTTGGGTGGTTATCTACCTAGTATGA-3′, S-INDEL reverse primer 5′-AAACGGCTCCTGTGAAATGC-3′, S-INDEL probe 5′-VIC/ATTTTCCTYAGTTACATCGAT/MGB-3′. PCR reaction setup and amplification conditions are the same as for the aforementioned PEDV N gene-based real-time RT-PCR. The results were analyzed using an automatic baseline setting with a threshold at 0.1 for both non-S-INDEL PEDV and S-INDEL PEDV, with C_T_ values < 40 being considered positive.

### 2.5. Antibody Measurement by PEDV Whole Virus-Based ELISA

A previously described PEDV whole virus-based indirect ELISA [28,31] was used for IgG and IgA antibody testing in this study. Specifically, the US PEDV non-S-INDEL strain USA/IN19338/2013 was used in the PEDV whole virus-based antibody ELISA. Each batch of one liter of PEDV propagated in Vero cells (infectious titers ranging from 10^5^–10^6^ TCID50/mL) was subjected to one freeze–thaw and then centrifuged at 4000× *g* for 15 min to remove cell debris. The virus was then pelleted by ultracentrifugation at 140,992× *g* for 3 h. The virus pellet was washed twice with sterile PBS (1× pH 7.4) and then resuspended in PBS at a ratio of 1:100 of the original volume and stored at −80 °C. Polystyrene 96-well microtitration plates (Thermo Fisher Scientific) were coated with the viral antigen solution (100 μL/well) and incubated at 4 °C overnight. Plates were washed 5 times, blocked (300 μL/well) with PBS containing 1% bovine serum albumin (Jackson ImmunoResearch Inc., West Grove, PA, USA), and incubated at 25 °C for 2 h. Plates were then dried at 37 °C for 4 h and stored at 4 °C in a sealed bag with desiccant packs until use.

Serum, colostrum and milk samples were 1:50 diluted and added to the coated plates (100 μL/well). Plates were incubated at 25 °C for 1 h and then washed 5 times with PBST wash solution (PBS 1×, 0.1% Tween-20, pH 7.4). Subsequently, 100 μL of peroxidase-conjugated goat anti-pig IgG (Fc) antibody (Bethyl Laboratories Inc., Montgomery, TX, USA) at 1:20,000 dilution for serum and colostrum/milk samples or goat anti-pig IgA (Bethyl Laboratories Inc.) at 1:3000 dilution for serum samples and 1:45,000 dilution for colostrum/milk samples was added to each well, and the plates were incubated at 25 °C for 1 h. After a washing step, 100 μL of tetramethylbenzidine-hydrogen peroxide substrate solution (TMB, Dako North America Inc., Carpinteria, CA, USA) was added to each well. The plates were incubated for 5 min at room temperature, and the reaction was stopped by adding 50 μL of stop solution (1 M sulfuric acid) to each well. Reactions were measured as optical density (OD) at 450 nm using an ELISA plate reader operated with commercial software (Biotek^®^ Instruments Inc., Winooski, VT, USA). The antibody response in serum and colostrum/milk samples was represented as sample-to-positive (S/P) ratios calculated as: S/P ratio = (sample OD − blank well control mean OD)/(positive control mean OD − blank well control mean OD).

For serum IgG and IgA, the S/P ratio of 0.7 was tentatively used for analyses in this study with an S/P ratio ≥ 0.7 being considered as positive and S/P ratio < 0.7 being considered as negative. No S/P ratio cutoffs have been established for colostrum/milk samples, but in this study, the S/P ratio of 0.6 was tentatively used for analyses of PEDV IgG and IgA in colostrum/milk samples.

### 2.6. Statistical Analysis

Generalized linear mixed (GLIMMIX) model was used for statistical analyses with Statistical Analysis System (SAS) version 9.3 (SAS institute, Cary, NC, USA). Comparisons at each time point between different groups were conducted using Graph Pad software. *p* value < 0.05 was defined as statistically significant.

## 3. Results

### 3.1. Infectious Titers of Virus Inocula

The PEDV MLV product IVP (lot # K1115LS22), derived from the S-INDEL PEDV isolate USA/IL20697/2014, was expected to have an infectious titer of 10^4^ TCID50/mL. Each gilt in the T01 V/C group was orally inoculated with 10 mL virus inoculum with an expectation of 10^5^ TCID50 of virus at each vaccination. Back titration of inoculum after the first vaccination revealed a titer of 1.5 × 10^3^ TCID50/mL. Similarly, back titration of inoculum after the second vaccination indicated a titer of 7.4 × 10^2^ TCID50/mL. This suggests that each gilt in the T01 V/C group actually received 1.5 × 10^4^ TCID50 at the first vaccination and 7.4 × 10^3^ TCID50 at the second vaccination.

Back titration of the challenge virus non-S-INDEL PEDV USA/IN19338/2013 P7 revealed that the inoculum had a titer of 3 × 10^2^ TCID50/mL. Since each piglet was given 2 mL, the total amount actually received was 6 × 10^2^ TCID50/piglet.

### 3.2. Clinical Observations of Gilts after Vaccination but before Piglet Challenge

No abnormal clinical signs (e.g., diarrhea, depression, dehydration, etc.) were observed in any gilt treatment group after the first vaccination at 79 days of gestation or when boostered at 100 days of gestation.

### 3.3. PEDV Fecal Shedding in Gilts

**Figure 2:**
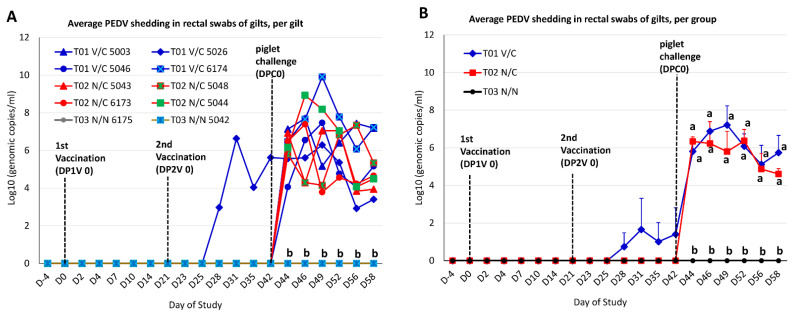
PEDV shedding in rectal swabs of gilts as determined by a quantitative PEDV N gene-based RT-PCR. (**A**) The virus titers in individual gilts at each time point. (**B**) The virus titers at each time point were the mean values of the gilts (both PCR positive and PCR negative gilts) in each group. Different letters indicate significant differences.

Before piglet challenge, all gilt rectal swabs from T02 N/C and T03 N/N groups at D-4 to D42 were negative by PEDV real-time RT-PCR (Figure 2). Among the T01 V/C group, three gilts (#5003, #5046 and #6174) did not have detectable virus in rectal swabs as tested by PEDV real-time RT-PCR, after two vaccinations through D42 before piglet challenge; in contrast, gilt #5026 shed PEDV at D28, D31, D35 and D42 with 10^2.9^, 10^6.6^, 10^4.0^, and 10^5.6^ genomic copies/mL, respectively. A spike gene-based differential real-time RT-PCR, which can distinguish the US non-S-INDEL and S-INDEL PEDV strains, confirmed that the PEDV detected from the rectal swabs of gilt T01 V/C #5026 through D42 (before piglet challenge) was the S-INDEL PEDV strain.

After piglet challenge, gilts remained on test until D58 when piglets were 21 days old (DPC 16). As expected, T03 N/N gilt rectal swabs from DPC 0 to DPC 16 (D42−D58) had no detectible virus by PEDV PCR (Figure 2). All four T01 V/C gilts and four T02 N/C gilts started shedding PEDV from D44 (DPC 2) through D58 (DPC 16). While there were no significant differences in rectal swab viral shedding levels between T01 V/C and T02 N/C on average (Figure 2B), there was considerable variation within these groups (Figure 2A).

A spike gene-based PEDV differential real-time RT-PCR revealed that a rectal swab from T01 V/C gilt #5026 at D44 (DPC 2) was positive for US S-INDEL PEDV (C_T_ 33.6) and US non-S-INDEL PEDV (C_T_ 34.9), indicating that a residual vaccine virus and challenge virus were detected in this sample. However, all PEDV positive swabs from remaining T01 V/C gilts and all T02 N/C gilts contained only the challenge virus (non-S-INDEL PEDV).

### 3.4. Antibody Responses of Gilts

PEDV IgG and IgA antibody responses in serum samples of gilts after vaccination (during gestation) and after piglet challenge through D58 (gilt necropsy, DPC 16) are summarized in Table 2. The serum samples of two gilts in the T03 N/N group were PEDV IgG and IgA antibody negative from D0 through D58 except at two time points (D0 and D14), at which the two gilts had IgG S/P ratio directly above the cutoff values but at which IgA antibody was clearly negative. From D0 (first vaccination) to D42 (before piglet challenge), all gilt serum samples collected from the T02 N/C group were PEDV IgG and IgA antibody negative. After piglet challenge, 3/4 and 1/4 gilts in the T02 N/C group were serum PEDV IgG and IgA antibody positive at D49 (DPC 7), respectively, whereas all four gilts in T02 N/C were serum PEDV IgG and IgA antibody positive at D58 (DPC 16). Among the T01 V/C group, serum samples from three of four gilts (#5003, #5046, and #6174) remained PEDV IgG and IgA antibody negative from D0 to D42, but they gradually became PEDV IgG and IgA antibody positive at D49 (DPC 7) and/or D58 (DPC 16). Serum samples of gilt #5026 were PEDV IgG and IgA antibody negative from D0 to D28 but were PEDV IgG and IgA antibody positive at D35 (partition) and at D42 (DPC 0) through termination of the study at D58 (DPC 16). There were no significant differences between the T01 V/C group and the T02 N/C group at D42 (DPC 0), D49 (DPC 7) and D58 (DPC 16) in terms of the average PEDV IgG and PEDV IgA antibody levels in serum samples (Table 2).

Gilt colostrum/milk antibody responses as tested by PEDV whole virus-based ELISA are summarized in Table 3. Colostrum/milk samples collected from the T03 N/N group were PEDV IgG and IgA antibody negative throughout the study. In the T02 N/C group, colostrum/milk samples from all gilts were PEDV IgG antibody negative from D42 (DPC 0) through D49 (DPC 7) but 2/3 gilts and 3/3 gilts were PEDV IgG antibody positive at D52 (DPC 10) and D56 (DPC 14), respectively. Regarding the PEDV IgA antibody in colostrum/milk samples of the T02 N/C group, all gilts were negative from D42 (DPC 0) to D52 (DPC 10) and only 2/3 gilts were positive at D56 (DPC 14). Colostrum/milk samples from gilts in the T01 V/C group were PEDV IgG and IgA antibody negative at D42 (DPC 0) except that the gilt T01 V/C 5003 had slight positive PEDV IgG antibody. From D42 to D49 (DPC 2 to DPC 7), only the milk samples from the gilt T01 V/C 5026 were PEDV IgG and IgA antibody positive; the milk sample from the gilt T01 V/C 5046 was also PEDV IgG antibody positive at D49 (DPC 7). From D52 to D56 (DPC 10 to DPC 14), all gilt milk samples in the T01 V/C group were PEDV IgG antibody positive while only 2/4 and 3/4 gilts were PEDV IgA antibody positive in their milk samples at D52 (DPC 10) and D56 (DPC 14), respectively. There were no significant differences between the T01 V/C and the T02 N/C groups from D42 to D56 (DPC 0 to DPC 14) in regard to the average PEDV IgG antibody and PEDV IgA antibody levels in their colostrum/milk samples.

### 3.5. Piglet Mortality before and after PEDV Challenge

Gilts farrowed within three days of each other. Table 4 summarizes live and dead piglets before and after PEDV challenge. Initially, 107 live piglets were farrowed by the ten gilts. However, six piglet mortalities (four from T01 V/C and two from T02 N/C) occurred via laid-on-by-dam before piglet challenge day. Overall, 44 live piglets from T01 V/C, 39 live piglets from T02 N/C, and 18 live piglets from T03 N/N were challenged with PEDV.

**Figure 3:**
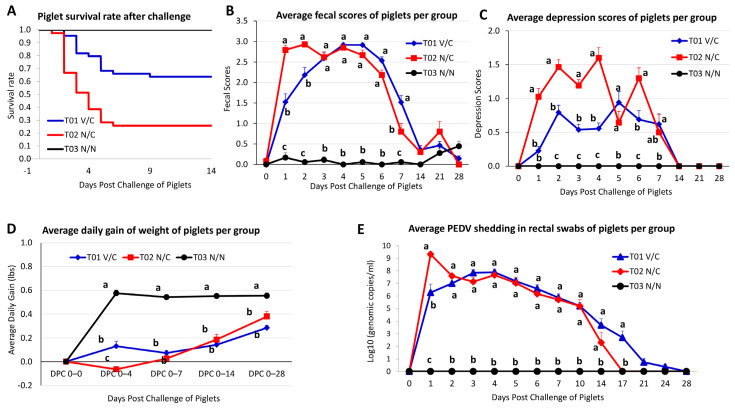
The survival rate, clinical signs, average daily gain, and viral shedding in piglets from the three experimental groups. Pregnant sows were orally vaccinated 5-weeks pre-farrowing and then boosted three weeks later. Piglets were challenged with virulent non-S-INDEL PEDV at 3 to 5 days of age. (**A**) Survival rate of piglets. (**B**) Average fecal score for diarrheal severity over time. (**C**) Average depression scores over time. (**D**) Mean average daily weight gain after challenge. Only piglets that survived through DPC 28 were included for weight comparison. (**E**) PEDV RNA titers in rectal swabs as determined by a quantitative PEDV N gene-based real-time RT-PCR. Virus titers represent the mean values of all available piglets in each group at each time point. For each figure, error bars indicate standard error, and different letters indicate significant differences.

After challenge, piglet mortalities from T01 V/C, T02 N/C, and T03 N/N groups were 36.4% (16/44), 74.4% (29/39), and 0% (0/18), respectively (Table 4). Piglet #47 in the litter of gilt #5046 was laid on by dam to death; if this piglet were not counted as death due to PEDV, the piglet mortality from T01 V/C group would be 34.1%. Among the four litters in the T01 V/C group, there were 2, 6, 1, 5, 1, 0, 0, and 1 piglet deaths per day from DPC 2 to DPC 9, respectively. Among the four litters in the T02 N/C group, there were 1, 12, 6, 5, 4 and 1 piglet deaths per day from DPC 1 to DPC 6, respectively. In both groups, most piglet deaths occurred during DPC 2−5 (Table 4 and Figure 3A). During DPC 10−28, there were no piglet deaths in either the T01 V/C or the T02 N/C group.

Conversely, there were variations in piglet mortality between litters. For example, the T01 V/C piglet mortalities were 0% (the litter of gilt #5026), 30% (the litter of gilt #6174), 55.6% (the litter of gilt #5046) and 72.7% (the litter of gilt 5003). The T02 N/C piglet mortalities were 22.2% (the litter of gilt #5043), 81.8% (the litter of gilt #5048), 92.9% (the litter of gilt #6173) and 100% (the litter of gilt 5044).

### 3.6. Clinical Effects in Post-Challenged Piglets

To evaluate clinical effects in post-challenged litters, diarrhea and depression levels were scored, average daily gain was calculated, and virus shedding was evaluated, which are summarized in Figure 3. As expected, T03 N/N piglets had normal or rarely transient soft feces during DPC 0 to DPC 28 (Figure 3B). Nearly all piglets from T02 N/C developed watery diarrhea starting from DPC 1 and continuing through DPC 5−6; diarrhea became milder from DPC 7 to the end of study. Piglets from T01 V/C gilt #6174 had diarrhea that trended similarly to those of the T02 N/C group. Piglets from T01 V/C gilts #5003 and #5046 developed soft to liquid (with some content) diarrhea at DPC 1 that progressed to watery diarrhea from DPC 2, lasting through DPC 5−6; diarrhea became milder from DPC 7 and thereafter. The litter from T01 V/C gilt #5026 lacked diarrhea at DPC 1, had mild diarrhea at DPC 2, and then watery diarrhea from DPC 3 lasting through DPC 5−6; diarrhea became milder from DPC 7 and thereafter. When the average fecal scores of piglets were compared between groups, the T01 V/C and T02 N/C groups had significantly higher fecal scores than T03 N/N during DPC 1−7 (*p* < 0.0001); T02 N/C group had significantly higher fecal scores than the T01 V/C group at DPC 1 (*p* < 0.0001) and DPC 2 (*p* < 0.0001) (Figure 3B). Clinical depression, dehydration, and appetite scores were also monitored. As expected, piglets from N/N lacked depression, dehydration, or inappetence. In contrast, depression, dehydration, and inappetence were observed to various degrees in T01 V/C and T02 N/C litters. Overall, T01 V/C piglets had significantly lower depression scores from DPC 1 to DPC 6 (*p* < 0.0001 to *p* < 0.0003), less severe dehydration from DPC 2 to DPC 4, and less severe loss of appetite from DPC 1 to DPC 4 as compared to T02 N/C. As exemplified in Figure 3C, the T03 N/N piglets did not show depression from DPC 0−28, whereas depression was observed in both T01 V/C and T02 N/C piglets during DPC 1−7. However, the depression scores of T02 N/C piglets were significantly higher than those of T01 V/C piglets at DPC 1−4 (*p* < 0.0001) and DPC 6 (*p* < 0.0003) (Figure 3C).

To evaluate clinical effects of PEDV-associated diarrhea in neonatal piglets, the mean value of body weight in each group was calculated during designated intervals as average daily gain (ADG): DPC 0−4, DPC 0−7, DPC 0−14, and DPC 0−28, which are summarized in Figure 3D. For the T02 N/C group, the litter of gilt #5044 had no surviving piglets through DPC 28, and the litter of gilt #6173 had one piglet that survived through DPC 28, these two litters were not included for comparison because statistical analyses could not be appropriately performed on these two litters. The T03 N/N group had significantly higher ADGs compared to the T01 V/C and T02 N/C groups regardless of when ADGs were calculated during intervals of DPC 0−4, 0−7, 0−14, or 0−28. During DPC 0−4, the T01 V/C group had a positive mean ADG of 0.13 pounds that was significantly higher than the ADG (−0.06 pounds) of the T02 N/C group. During DPC 0−7, 0−14, or 0−28, both the T01 V/C and the T02 N/C groups had positive mean ADGs, but differences were not significant between the two groups.

The changes and duration of fecal PEDV shedding in rectal swabs of challenged piglets were determined using a PEDV N gene-based RT-qPCR and are presented as mean values in Figure 3E. All rectal swabs from T03 N/N piglets lacked detectible PEDV for the study duration (DPC 0−28). Except for piglets from gilt T01 V/C #5026, all piglets from T01 V/C and T02 N/C groups were PEDV PCR positive at DPC 1. The piglets from gilt T01 V/C #5026 had delayed onset of viral shedding in rectal swabs (0/14 positive at DPC 1 and 10/14 positive at DPC 2) as compared to the other piglets from T01 V/C group or T02 N/C group, but all piglets in litter T01 V/C #5026 eventually shed virus starting from DPC 3. The mean genomic copies per milliliter of virus shed in rectal swabs were significantly higher in the T02 N/C group than in T01 V/C at DPC 1, but there were no significant differences between the two groups during DPC 2−14. All surviving piglets (10/10) from the T02 N/C group stopped shedding virus in rectal swabs from DPC 17. Most piglets (23/28) from T01 V/C litters stopped shedding virus in rectal swabs from DPC 21, although a few piglets (5/28) still shed low amounts of virus (high C_T_ values) at DPC 21 and DPC 24; all piglets from T01 V/C litters completely stopped shedding virus in rectal swabs at DPC 28.

As aforementioned, gilt T01 V/C #5026 was the only gilt that shed PEDV in rectal swabs after vaccination with S-INDEL strain and before challenge with non-S-INDEL PEDV at D42 (DPC 0). To determine if piglets from gilt T01 V/C #5026 acquired any S-INDEL PEDV from their dam, rectal swabs of piglets from gilt T01 V/C #5026 collected at DPC 0−3 were tested by a PEDV S gene-based differential PCR. For those PCR-positive rectal swabs, only the challenge virus non-S-INDEL PEDV was detected in them, and none of the samples were positive for S-INDEL PEDV, suggesting that the piglets from gilt T01 V/C #5026 did not acquire S-INDEL PEDV from their dam.

### 3.7. Antibody Responses in Piglets before and Post-PEDV Challenge

**Figure 4:**
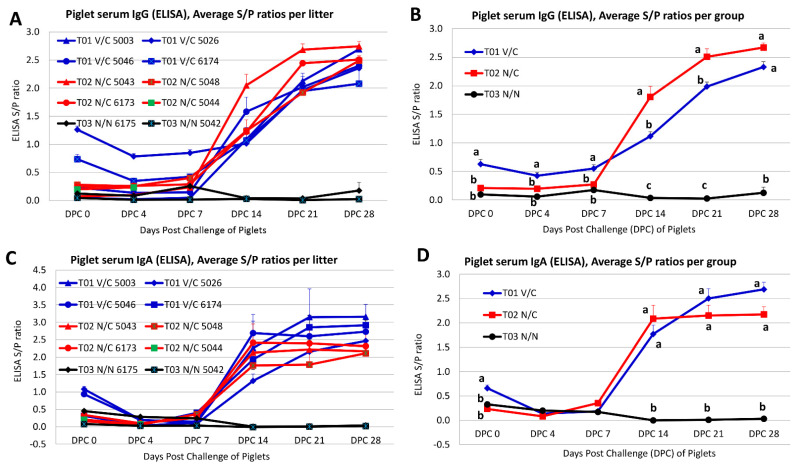
The S/P ratios of PEDV antibody in piglet sera as tested by a PEDV whole virus-based ELISA. (**A**) The average S/P ratios of piglet serum IgG per litter. (**B**) The average S/P ratios of piglet serum IgG per group. (**C**) The average S/P ratios of piglet serum IgA per litter. (**D**) The average S/P ratios of piglet serum IgA per group. Error bars indicate standard error. Different letters indicate significant differences. The positive cutoff for serum IgG and IgA was an S/P ratio equal to or greater than 0.7.

To evaluate piglet serum IgG and IgA antibody responses, a whole virus-based ELISA was performed. Mean serum IgG (Figure 4A) and IgA (Figure 4C) levels per litter and mean serum IgG (Figure 4B) and IgA (Figure 4D) levels per group were measured at DPC 0, 4, 7, 14, 21, and 28. As expected, the serum samples collected from piglets in the T03 N/N group were PEDV IgG and IgA antibody negative.

As shown in Figure 4A, piglets from T01 V/C #5003 and T01 V/C #5046 remained serum PEDV IgG antibody negative during DPC 0−7, but serum PEDV IgG antibody had a sharp increase from DPC 14 and remained positive through DPC 28. Piglets of T01 V/C #6174 were serum PEDV IgG antibody positive at DPC 0, negative during DPC 4−7, and positive again from DPC 14 through DPC 28. Piglets of T01 V/C #5026 were serum PEDV IgG antibody positive throughout the study DPC 0−28 but with increasing IgG antibody titers starting from DPC 14. Four litters of piglets in the T02 N/C group were consistently PEDV IgG antibody negative in their serum samples during DPC 0−7; however, all surviving piglets in this group had a sharp increase in PEDV IgG antibody titers from DPC 14 (Figure 4A). When the average piglet serum PEDV IgG antibodies were compared between groups, the T01 V/C group had significantly higher (*p* < 0.0005) PEDV IgG antibody titers in piglet serum samples than the T02 N/C group from DPC 0−7 (Figure 4B). However, the surviving piglets in the T02 N/C group developed significantly higher (*p* < 0.0001) serum PEDV IgG antibody titers than the T01 V/C group from DPC 14 to DPC 21 (Figure 4B).

As shown in Figure 4C, piglets from T01 V/C #5003 and T01 V/C #6174 remained serum PEDV IgA antibody negative during DPC 0−7, but serum PEDV IgA antibody had a sharp increase from DPC 14 and remained positive through DPC 28. Piglets from T01 V/C #5026 and T01 V/C #5046 were PEDV IgA positive at DPC 0, negative during DPC 4−7, and positive again from DPC 14 through DPC 28. Four litters of piglets in the T02 N/C group were consistently PEDV IgA negative in their serum samples during DPC 0−7; however, all surviving piglets in this group had a sharp increase in PEDV IgA antibody titers from DPC 14 (Figure 4C). When the average piglet serum PEDV IgA antibodies were compared between groups, the T01 V/C group had significantly higher (*p* < 0.0001) PEDV IgA antibody titers in piglet serum samples than the T02 N/C group at DPC 0 (Figure 4D). The differences in PEDV IgA antibody titers in piglet serum samples were not significant between the T01 V/C and T02 N/C groups from DPC 4 to DPC 28 (Figure 4D).

## 4. Discussion

PEDV continues to cause significant economic losses in the swine industry. Among the two major PEDV strains circulating in US swine (non-S-INDEL and S-INDEL), the S-INDEL PEDV strain caused noticeably milder clinical signs based on field observations in US swine herds [10]. However, an outbreak of S-INDEL PEDV in Germany (99.4% homology to US S-INDEL OH851 strain) reported varied clinical signs, including higher than expected mortalities in suckling piglets on one of the farms [32]. Nonetheless, the US S-INDEL PEDV was confirmed to be less pathogenic than the US non-S-INDEL PEDV strain in young piglets under experimental conditions [11,13]. In addition, it appears that S-INDEL PEDV could provide partial protection against the virulent non-S-INDEL PEDV [13,17]. All of these data suggest that S-INDEL may be a suitable live-attenuated PEDV vaccine candidate, although more studies are needed; for example, experimental vaccination of pregnant gilts/sows with S-INDEL PEDV followed by evaluation of its protective efficacy in piglets challenged with virulent non-S-INDEL PEDV has not been conducted. This study aimed to answer this question.

In the present study, gilts were orally vaccinated 5 weeks prior to parturition (the second trimester stage) and again 2 weeks prior to farrowing (the third trimester stage) to provide enough time for the development of protective lactogenic immunity against challenge. This design is in agreement with a study that demonstrated that immunization of gilts with PEDV at the second trimester induces higher antibody responses and better protection of piglets against challenge [33]. The experimental PEDV vaccine product IVP (lot #K1115LS22) derived from the S-INDEL isolate USA/IL20697/2014 was safe in pregnant gilts when orally administered 5 and 2 weeks prior to farrowing. No clinical signs (e.g., diarrhea, depression, dehydration, etc.) were observed in any gilts after the first or second administration. However, among the four inoculated gilts (#5003, 5026, 5046, and 6174) in the T01 V/C group, only one gilt #5026 shed PEDV in rectal swabs from D28 to D42 before piglet challenge (Figure 2), suggesting active replication and shedding of the vaccine virus in this gilt. As a result, this was the only gilt from the group that had anti-PEDV IgG and IgA antibodies consistently detected in her serum and colostrum/milk samples before the challenge virus induced antibodies in gilts (Table 2 and Table 3). The other three gilts in the T01 V/C group did not shed the vaccine virus in rectal swabs prior to piglet challenge (DPC 0, Figure 2), and no anti-PEDV IgG or IgA antibodies were consistently detected in serum and colostrum/milk samples before the challenge virus induced antibodies in gilts (Table 2 and Table 3). All gilts in the T01 V/C and T02 N/C groups started to shed virus two days post challenge of their piglets (Figure 2), suggesting that infection of piglets resulted in contact exposure of gilts via fecal virus shedding. This further induced anti-PEDV IgG and IgA antibodies in gilt serum samples one-week post challenge and in gilt colostrum/milk samples 10−14 days post challenge (Table 2 and Table 3).

Once farrowed, piglets were kept together with their birth dams to nurse. All piglets in the T02 N/C and T03 N/N groups were PEDV IgG and IgA antibody negative in their serum samples before challenge. Among the T01 V/C group, there was a large variability of piglet serum IgG/IgA levels between litters before challenge. Two litters from T01 V/C gilts #5026 and #6174 had positive serum PEDV IgG at DPC 0; two litters from T01 V/C gilts #5026 and #5046 had positive serum PEDV IgA at DPC 0 (Figure 4). This discrepancy is likely attributed to the differences of maternal antibodies acquired from the dams’ colostrum/milk. All survived piglets in the T01 V/C and T02 N/C groups later on developed PEDV IgG and IgA antibodies induced by the challenge virus (Figure 4).

A large variation in overall mortality after piglet challenge was also observed between litters across T01 V/C and T02 N/C groups. In the T01 V/C group, mortalities of 72.7%, 55.6%, 30.0%, and 0% were observed at the litter level with an average mortality of 36.4% at the group level. In the T02 N/C group, piglet mortality rates were 100%, 92.9%, 81.8% and 22.2% at the litter level with an average mortality of 74.4% at the group level. In general, it has been reported that death rates average 50% in suckling piglets up to one week of age (often approaching 100% in neonates less than 3 days old) that subsequently decrease to 10% [1]. Likewise, our study reproduced high mortality in T02 N/C piglets in all but one litter (gilt #5043). Piglets in this litter eventually developed numerically higher serum IgG than the other litters even including the T01 V/C group, but piglet serum anti-PEDV IgA levels were similar across litters (Figure 4). Serum PEDV IgG antibody has a systemic protective role as suggested by a previous study [27]. Among the T01 V/C group, variations in higher than expected mortality across litters could be associated with incomplete activation of the vaccinated dams’ gut mucosal immune system; none of these gilts had consistently detected serum and/or colostrum/milk antibodies, except for gilt #5026 who developed consistent anti-PEDV IgG and IgA antibodies and had 0% litter mortality. The survived piglets in the T01 V/C and T02 N/C groups were monitored through DPC 28 for shedding of the challenge virus in rectal swabs. Comparison of PEDV fecal shedding results in piglets of T01 V/C and T02 N/C appears to suggest that gilt immunization (T01 V/C group) did not significantly shorten the duration of challenge virus fecal shedding in offspring (Figure 3E).

When all of the data (vaccine virus shedding in gilts, PEDV antibodies in gilt sera and colostrum/milk samples after vaccination but before piglet challenge, PEDV antibodies in piglet sera before challenge, piglet mortality, and piglet virus shedding) were taken together for analyses, it was evident that gilt T01 V/C 5026 actively replicated and shed the vaccine virus, consistently developed anti-PEDV IgG and IgA in gilt sera and IgG in colostrum/milk samples before piglet challenge, had consistent IgG and IgA antibodies in their piglets before challenge, had 0% mortality, and had delayed onset of virus shedding by piglets. In addition, even at the group level, gilt immunization with S-INDEL PEDV had several benefits such as significantly lower fecal scores in the first 48 h, significantly greater ADG in the first four days post challenge, and decreased piglet mortality as compared to the T02 N/C group. These results indicate that partial cross-protection in piglets of the T01 V/C group was achieved.

It was initially planned to orally inoculate each gilt with 10^5^ TCID50 of the attenuated PEDV MLV product IVP derived from an S-INDEL isolate at each vaccination time point. However, the back titration indicated that each gilt in the T01 V/C group actually received 1.5 × 10^4^ TCID50 and 7.4 × 10^3^ TCID50 of the vaccine virus at the first and the second administration, respectively. A previous study demonstrated that the minimum infectious doses for a US non-S-INDEL PEDV strain are 0.56 TCID50 to infect naïve 5-day-old piglets and 56 TCID50 to infect 3-week-old pigs [28]. It is unknown what the minimum infectious dose is for the US S-INDEL PEDV to infect pregnant females and what the infectious dose is for the US S-INDEL PEDV to establish infection in most inoculated pregnant females. We hypothesize that if S-INDEL PEDV can be grown to higher infectious titers and induce active replication in pregnant gilts/sows, PEDV-specific protective immune responses are likely to be elicited in pregnant gilts/sows, and such immune responses may efficiently protect the piglets from challenge with a virulent US non-S-INDEL PEDV strain. This warrants further investigations in future studies. However, the difficulty to propagate the S-INDEL PEDV to high infectious titer could be one challenge. In addition, based on this study, it is evident that variation between gilts and litters are present, and a larger number of gilts/sows shall be included in future studies.

## 5. Conclusions

In summary, our study describes a vaccine-challenge experiment using an oral prime and boost regiment in gestating gilts with an attenuated US S-INDEL PEDV strain. Protective lactogenic immunity was achieved in one litter when challenged with virulent US PEDV non-S-INDEL isolate USA/IN19338/2013. Thus, cell-cultured adapted S-INDEL USA/IL20697/2014 isolate could be a potential live-attenuated PEDV vaccine candidate if higher infectious titer can be obtained and if the virus can efficiently replicate in inoculated pigs to induce adequate immune responses.

## Figures and Tables

**Table 1:** Experimental design of vaccination and challenge schedules.

Group	Number of Gilts/Litters	1st Vaccination (79 Days of Gestation)	2nd Vaccination (100 Days of Gestation)	Piglet Challenge	Wean Piglets	Piglet Necropsy
T01 (V/C)	4	PEDV vaccine candidate (IVP): target titer of 10^4^ TCID50/mL, 10 mL per gilt, oral	PEDV vaccine candidate (IVP): target titer of 10^4^ TCID50/mL, 10 mL per gilt, oral	T01 and T02: piglets challenged with virulent non-S-INDEL PEDV on the same day when piglets at 3 ± 2 days of age, 5 × 10^2^ TCID50/mL, 2 mL per piglet, oral	T01, T02, and T03: when piglets were ~21 days old, gilts were euthanized and piglets were weaned	T01, T02, and T03: when piglets died; when piglets met euthanasia criteria; remaining live piglets at 28 days post challenge
			
T02 (N/C)	4	Media Control (CP): 10 mL per gilt, oral	Media Control (CP): 10 mL per gilt, oral			
				
T03 (N/N)	2	Media Control (CP): 10 mL per gilt, oral	Media Control (CP): 10 mL per gilt, oral	T03: piglets challenged with virus-negative medium, 2 mL per piglet, oral		

Note: V/C: vaccination/challenge; N/C: non-vaccination/challenge; N/N: non-vaccination/non-challenge.

**Table 2:** Gilt anti-PEDV IgG and IgA response in serum as tested by PEDV whole virus-based ELISA (S/P ratios shown in the table).

Antibody Isotype	Group	Gilt ID	DP1V 0	DP1V 7	DP1V 14	DP2V 0	DP2V 7	Farrow	DPC 0	DPC 7	DPC 16
			D0	D7	D14	D21	D28	D36–D39	D42	D49	D58 *
IgG	T01	5003	0.5411	0.6365	0.6584	0.5189	0.0329	0.0209	0.0269	0.2749	2.3297
	V/C	5026	0.1256	0.1683	0.1663	0.0867	0.0886	1.3576	3.1444	2.9761	3.2600
		5046	0.3237	0.4771	0.4602	0.3187	0.1614	0.3675	0.6325	2.9622	3.1275
		6174	0.3353	0.5777	0.7291	0.4890	0.3546	0.2918	0.2231	3.3088	3.4014
		Average	0.3314	0.4649	0.5035	0.3533	0.1594	0.5095	1.0067 ^a^	2.3805 ^a^	3.0297 ^a^
										
	T02	5043	0.3836	0.7440	0.6580	0.6454	0.1237	0.1111	0.0435	0.3884	2.8000
	N/C	5048	0.3092	0.5604	0.4734	0.4715	0.2415	0.2135	0.1188	1.0966	2.8464
		6173	0.5662	0.6618	0.5894	0.5710	0.2435	0.1585	0.2628	0.8058	2.9111
		5044	0.5507	0.5130	0.3150	0.4280	0.1208	0.1903	0.1575	1.0763	2.3353
		Average	0.4524	0.6198	0.5090	0.5290	0.1824	0.1684	0.1457 ^a^	0.8418 ^a^	2.7232 ^a^
										
	T03	6175	0.8609	0.6300	0.8213	0.6106	0.1246	0.0918	0.0986	0.1063	0.0928
	N/N	5042	0.9594	0.6174	0.9488	0.5758	0.1266	0.0454	0.0879	0.0551	0.1913
		Average	0.9102	0.6237	0.8851	0.5932	0.1256	0.0686	0.0933 ^a^	0.0807 ^a^	0.1421 ^b^
IgA	T01	5003	0.0949	0.1227	0.1031	0.1332	0.0796	0.0561	0.0326	0.4060	4.4883
	V/C	5026	0.0437	0.0757	0.1018	0.0679	0.1906	0.8251	2.7624	2.6057	3.6632
		5046	0.1436	0.1384	0.1410	0.0731	0.1214	0.5718	0.2154	1.5953	2.4047
		6174	0.0424	0.0509	0.0809	0.0666	0.0731	0.0692	0.0313	0.2415	1.6893
		Average	0.0812	0.0969	0.1067	0.0852	0.1162	0.3806	0.7604 ^a^	1.2121 ^a^	3.0614 ^a^
										
	T02	5043	0.0587	0.1174	0.0861	0.1061	0.0387	0.0687	0.0225	0.3870	3.3620
	N/C	5048	0.0300	0.0462	0.0350	0.0474	0.0424	0.0474	0.0300	0.1598	2.7790
		6173	0.1099	0.0899	0.0662	0.0999	0.1785	0.1211	0.1461	0.3034	2.7665
		5044	0.0762	0.0624	0.0400	0.0787	0.0824	0.1548	0.0924	1.3408	3.6554
		Average	0.0687	0.0790	0.0568	0.0830	0.0855	0.0980	0.0728^a^	0.5478 ^a^	3.1407 ^a^
										
	T03	6175	0.3908	0.2709	0.4370	0.4295	0.1473	0.4732	0.2222	0.1461	0.0899
	N/N	5042	0.1523	0.0737	0.1673	0.0861	0.1273	0.0300	0.1248	0.0375	0.0512
		Average	0.2716	0.1723	0.3022	0.2578	0.1373	0.2516	0.1735 ^a^	0.0918 ^a^	0.0706 ^b^

Notes: V/C: vaccination/challenge; N/C: non-vaccination/challenge; N/N: non-vaccination/non-challenge. DP1V: day post 1st vaccination; DP2V: day post 2nd vaccination; DPC: day post challenge; D0-D58: day of study. * Gilts euthanized on D58. Different letters (a and b) indicate statistically significant difference of IgG between groups and IgA between groups at each time point D42, D49 and D58. The positive cutoff for serum IgG and IgA was an S/P ratio equal to or greater than 0.7, as indicated in red.

**Table 3:** Gilt anti-PEDV IgG and IgA response in colostrum/milk as tested by PEDV whole virus-based ELISA (S/P ratios shown in the table).

Antibody Isotype	Group	Gilt ID	D42	D44	D46	D49	D52	D56
			DPC 0	DPC 2	DPC 4	DPC 7	DPC 10	DPC 14
IgG	T01 V/C	5003	0.7903	0.5831	X	0.0166	0.8213	1.1731
		5026	0.0346	0.9321	1.1440	0.6856	0.6884	0.7091
		5046	0.0596	0.0568	0.0305	0.8089	1.5623	1.1413
		6174	0.0014	0.0055	0.2008	0.4474	3.2729	3.6745
		Average	0.2215 ^a^	0.3944 ^a^	0.4584 ^a^	0.4896 ^a^	1.5862 ^a^	1.6745 ^a^
							
	T02 N/C	5043	−0.0194	0.0097	0.0222	0.0443	0.4437	0.6971
		5048	0.0055	0.0180	−0.0069	0.1773	2.2995	2.8063
		6173	−0.0235	0.0097	0.0360	0.1316	0.8698	1.1515
		5044	0.0235	0.0014	0.0222	0.1510	X	X
		Average	−0.0035 ^a^	0.0097 ^a^	0.0184 ^a^	0.1261 ^a^	1.2043 ^a^	1.5516 ^a^
							
	T03 N/N	6175	0.0402	0.0000	0.0499	0.0360	0.0147	−0.0065
		5042	0.0055	−0.0028	0.0568	0.0817	0.0147	0.0374
		Average	0.0229 ^a^	−0.0014 ^a^	0.0534 ^a^	0.0589 ^a^	0.0147 ^a^	0.0155 ^a^
IgA	T01 V/C	5003	0.4527	0.3953	X	0.0721	0.1840	0.7342
		5026	0.1324	0.5735	0.6573	0.6446	0.8345	1.1091
		5046	0.1110	0.4197	0.2425	0.3359	0.8617	0.8111
		6174	0.0039	0.0662	0.1821	0.1071	0.1918	0.5034
		Average	0.1750 ^a^	0.3637 ^a^	0.3606 ^a^	0.2899 ^a^	0.5180 ^a^	0.7895 ^a^
							
	T02 N/C	5043	−0.0068	0.0302	0.0876	0.0380	0.1255	0.4649
		5048	−0.0049	0.0292	0.0263	0.1130	0.3385	0.8157
		6173	0.0010	0.1188	0.1402	0.3359	0.2592	0.6852
		5044	0.0156	0.0146	0.0769	0.0808	X	X
		Average	0.0012 ^a^	0.0482 ^b^	0.0828 ^a^	0.1419 ^a^	0.2411 ^a^	0.6553 ^a^
							
	T03 N/N	6175	0.0516	0.0166	0.0331	0.0380	0.0148	0.0033
		5042	0.0380	0.0282	0.1061	0.0935	0.0618	0.0493
		Average	0.0448 ^a^	0.0224 ^b^	0.0696 ^a^	0.0658 ^a^	0.0383 ^a^	0.0263 ^b^

Note: V/C: vaccination/challenge; N/C: non-vaccination/challenge; N/N: non-vaccination/non-challenge. DP1V: day post 1st vaccination; DP2V: day post 2nd vaccination; DPC: day post challenge; D0-D58: day of study. X: milk samples not available. Different letters (a and b) indicate statistically significant difference of IgG between groups and IgA between groups at each time point. The positive cutoff for colostrum IgG and IgA was an S/P ratio equal or greater than 0.6, as indicated in red.

**Table 4:** Summary of live and dead piglets at birth and after PEDV challenge.

Group	T01 V/C	T01 V/C	T01 V/C	T01 V/C	T02 N/C	T02 N/C	T02 N/C	T02 N/C	T03 N/N	T03 N/N
Gilt ID	#5003	#5026	#5046	#6174	#5043	#5048	#6173	#5044	#6175	#5042
Live born piglets	11	15	10	12	9	11	14	7	12	6
										
Dead piglets (laid by dam) prior to challenge	0	1	1	2	0	0	0	2	0	0
										
Number of piglets for challenge per litter	11	14	9	10	9	11	14	5	12	6
			
Number of piglets for challenge per group	44				39				18	
			
Challenge material	Non-S-INDEL PEDV USA/IN1938/2013				Non-S-INDEL PEDV USA/IN1938/2013				Virus-negative medium	
DPC 0: live (dead)	11	14	9	10	9	11	14	5	12	6
DPC 1: live (dead)	11	14	9	10	9	11	13 (1)	5	12	6
DPC 2: live (dead)	10 (1)	14	9	9 (1)	9	5 (6)	10 (3)	2 (3)	12	6
DPC 3: live (dead)	5 (5)	14	8 (1)	9	7 (2)	4 (1)	8 (2)	1 (1)	12	6
DPC 4: live (dead)	5	14	7 (1)	9	7	4	4 (4)	0 (1)	12	6
DPC 5: live (dead)	4 (1)	14	4 (3) *	8 (1)	7	2 (2)	2 (2)	0	12	6
DPC 6: live (dead)	3 (1)	14	4	8	7	2	1 (1)	0	12	6
DPC 7: live (dead)	3	14	4	8	7	2	1	0	12	6
DPC 8: live (dead)	3	14	4	8	7	2	1	0	12	6
DPC 9: live (dead)	3	14	4	7 (1)	7	2	1	0	12	6
DPC 10−28: live (dead)	3	14	4	7	7	2	1	0	12	6
										
Total: live (dead) piglets after challenge	3 (8)	14 (0)	4 (5)	7 (3)	7 (2)	2 (9)	1 (13)	0 (5)	12 (0)	6 (0)
										
Mortality per litter	72.7%	0.0%	55.6%	30.0%	22.2%	81.8%	92.9%	100.0%	0.0%	0.0%
Mortality per group	16/44 × 100% = 36.4%				29/39 × 100% = 74.4%				0/18 × 100% = 0%	

* Piglet #47 from the gilt #5046 was laid by dam to death on DPC 5. Here, this piglet was counted into post-challenge mortality.

## Data Availability

The raw data are available and can be provided upon request.
